# Dietary flaxseed administered post thoracic radiation treatment improves survival and mitigates radiation-induced pneumonopathy in mice

**DOI:** 10.1186/1471-2407-11-269

**Published:** 2011-06-24

**Authors:** Melpo Christofidou-Solomidou, Sonia Tyagi, Kay-See Tan, Sarah Hagan, Ralph Pietrofesa, Floyd Dukes, Evguenia Arguiri, Daniel F Heitjan, Charalambos C Solomides, Keith A Cengel

**Affiliations:** 1 Department of Medicine, Pulmonary Allergy and Critical Care Division, University of Pennsylvania Medical Center, Philadelphia, PA 19104, USA; 2 Biostatistics & Epidemiology, University of Pennsylvania Medical Center, Philadelphia, PA 19104, USA; 3 Radiation Oncology, University of Pennsylvania Medical Center, Philadelphia, PA 19104, USA; 4 Department of Pathology, Jefferson University Hospital, Philadelphia, PA 19140, USA

**Keywords:** Flaxseed, radiation pneumonopathy, mitigation, lung fibrosis, antioxidant, flaxseed lignans, SDG, lung injury, ROS, inflammation, bronchoalveolar lavage, survival, cytokines, mouse model

## Abstract

**Background:**

Flaxseed (FS) is a dietary supplement known for its antioxidant and anti-inflammatory properties. Radiation exposure of lung tissues occurs either when given therapeutically to treat intrathoracic malignancies or incidentally, such as in the case of exposure from inhaled radioisotopes released after the detonation of a radiological dispersion devise (RDD). Such exposure is associated with pulmonary inflammation, oxidative tissue damage and irreversible lung fibrosis. We previously reported that dietary FS prevents pneumonopathy in a rodent model of thoracic X-ray radiation therapy (XRT). However, flaxseed's therapeutic usefulness in mitigating radiation effects post-exposure has never been evaluated.

**Methods:**

We evaluated the effects of a 10%FS or isocaloric control diet given to mice (C57/BL6) in 2 separate experiments (n = 15-25 mice/group) on 0, 2, 4, 6 weeks post a single dose 13.5 Gy thoracic XRT and compared it to an established radiation-protective diet given preventively, starting at 3 weeks prior to XRT. Lungs were evaluated four months post-XRT for blood oxygenation levels, inflammation and fibrosis.

**Results:**

Irradiated mice fed a 0%FS diet had a 4-month survival rate of 40% as compared to 70-88% survival in irradiated FS-fed mouse groups. Additionally, all irradiated FS-fed mice had decreased fibrosis compared to those fed 0%FS. Lung OH-Proline content ranged from 96.5 ± 7.1 to 110.2 ± 7.7 μg/ml (Mean ± SEM) in all irradiated FS-fed mouse groups, as compared to 138 ± 10.8 μg/ml for mice on 0%FS. Concomitantly, bronchoalveolar lavage (BAL) protein and weight loss associated with radiation cachexia was significantly decreased in all FS-fed groups. Inflammatory cell influx to lungs also decreased significantly except when FS diet was delayed by 4 and 6 weeks post XRT. All FS-fed mice (irradiated or not), maintained a higher blood oxygenation level as compared to mice on 0%FS. Similarly, multiplex cytokine analysis in the BAL fluid revealed a significant decrease of specific inflammatory cytokines in FS-fed mice.

**Conclusions:**

Dietary FS given post-XRT mitigates radiation effects by decreasing pulmonary fibrosis, inflammation, cytokine secretion and lung damage while enhancing mouse survival. Dietary supplementation of FS may be a useful adjuvant treatment mitigating adverse effects of radiation in individuals exposed to inhaled radioisotopes or incidental radiation.

## Background

Ionizing radiation produces deleterious effects in living organisms. Rapid technological advancement has increased human exposure to ionizing radiations. People are exposed to ionizing radiation during diagnostic and therapeutic radiographic procedures, as well as in their daily activities at the work place [1]. Humans are also exposed to ionizing radiation during air and space travel, background radiation from nuclear accidents and through the use of electronic devices. Additionally, global developments of the past decade have established terrorism as a novel and highly concerning means by which large numbers of people could be exposed to potentially lethal amounts of radiation [2].

There are at least two potential ways that a terroristic attack could expose a population to radiation injury. If terrorists gained possession of a nuclear warhead, detonation could release large amounts of radiation (in a single "blast") that could induce radiation sickness, bone marrow damage and potential lung injury. More likely, however, the weapon of radiological terrorism would be a "dirty bomb", or a radiological dispersion device (RDD). In a RDD, conventional explosives would spread radioactive materials in the form of powder or pellets [2-4] which can spread far away from the immediate explosion and pose a high health risk if inhaled. Exposure to whole-body irradiation induces the well defined acute radiation syndrome (ARS), with symptoms from damage to hematopoietic, gastrointestinal and central nervous system [5]. However, in detonation of a RDD, the lung becomes the critical target organ for radiation injury.

Radiation pneumonopathy has been well characterized as a significant clinical toxicity from thoracic radiation [6,7]. Patients receiving large doses of radiation to the lung show two types of adverse clinical scenarios [8]. An acute type of radiation pneumonopathy can occur as early as two weeks after irradiation whereby a "pneumonitic" or exudative reaction occurs. In the second type of radiation-induced lung injury, occurring within several months after exposure, the lung tissue enters the "late fibrotic" phase, in which the number of inflammatory cells (particularly neutrophils) decrease and a marked thickening of alveolar walls due to collagen deposition can be noted histopathologically. Radiation pneumonopathy has been modeled in animals; the C57/BL6 mice are especially susceptible to this fibrotic reaction [9-11].

Several agents, ranging from cytokines to receptor blockers, have been tested for their efficacy in ameliorating radiation effects [10,12-14]. Unfortunately most agents, even those proven to be effective as radioprotectors (i.e., tested prior to a radiation exposure), are not yet available for human use. Additionally, these agents were intended as treatments for injuries resulting from the therapeutic use of radiation which is a very different scenario than radiation injuries resulting from nuclear accidents or radiological terrorism. Specifically, in most accident or terrorism scenarios, a) treatment would not be able to be initiated until after the irradiation, therefore, eliminating agents that work only when given before irradiation; b) the radiation would be received in a short overall time and there might be agents that are effective with multi-week radiation treatments that are less effective for a single large dose of radiation and c) a potential mitigator would need to be administered to a large population of healthy individuals exposed to an undetermined dose of radiation, making it highly desirable to find a agent that is non-toxic and safe for multiple administrations lasting throughout the long exposure and recovery phase.

High toxicity and unwanted side effects associated with chemical radioprotectors (thiols, antioxidant enzymes, etc) [15,16], has shifted the focus to the evaluation of the radioprotective potential of plants and herbs as well as antioxidant agents [17]. Our group has identified flaxseed and its bioactive lignan component as potent protectors against radiation-induced lung toxicity when given prior to radiation exposure [11]. Specifically, dietary flaxseed decreased radiation-induced oxidative lung tissue damage, decreased lung inflammation and prevented lung fibrosis. This study was performed to determine whether dietary flaxseed can also be effective as a mitigator of radiation toxicity, i.e., when administered after radiation exposure to the lung.

## Methods

### Animals

Our studies used female C57/BL6 mice, a strain well characterized in the field of pulmonary radioprotection [18,19]. Mice were obtained from Charles River (Wilmington, MA) and irradiated at 6-8 weeks of age under animal protocols approved by the Institutional Animal Care and Use Committee (IACUC) of the University of Pennsylvania. For this study we used n = 15 for each of the non-irradiated control groups (0%FS and 10%FS) and n = 25 for each of the irradiated mouse cohorts. The data shown here represent combined data from two separate studies.

### Diets and dietary treatments

**Figure 1:**
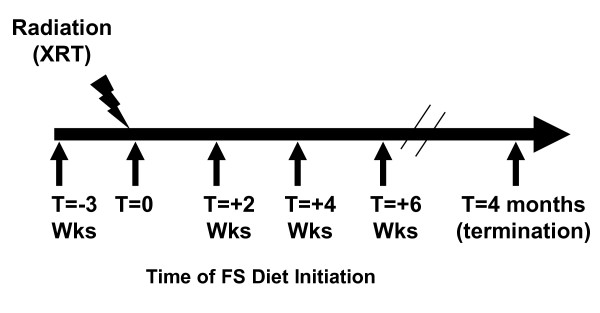
**Experimental Plan**. Mice were fed with 0% or 10%FS diet initiated prior (-3 weeks) or post (+2, +4, +6 weeks) single fraction (13.5 Gy) X-ray radiation therapy (XRT). Mice were sacrificed at 4 months post-XRT.

Semi-purified AIN-93G diet was used as the base diet which was supplemented with 10% (w/w) FS as described in our previous publications [11,20]. Control and experimental diets were isocaloric and identical in *Phys*siological fuel value. Whole ground yellow FS (Lot# 1012338) was kindly provided by Dr. James Hammond (North Dakota State University, Fargo, ND). Diets were started as described in Figure 1. Importantly, mice were maintained on the respective diets for the entire duration of the experiment until termination at 4 months post-radiation exposure.

### Radiation procedure

Mice were anesthetized and irradiated as previously described [10]. Briefly, using an immobilization chamber that allows bilateral exposure of the lung of up to 8 mice simultaneously while lead shielding (3 mm) the head, abdomen and extremities, 13.5 Gy was delivered to mid-plane using a 250 kVp orthovoltage machine (Philips RT 250) at a dose/rate of 1.7 Gy/min and a source to skin distance of 33 cm, through a 0.2 mm copper filter and a tube current of 13 mA. For quality assurance, thermoluminescent dosimeters were placed over selected mice to verify correct dose administration.

### Analytical evaluation of lignan content in murine plasma

Circulating plasma levels of the flaxseed lignans ED and EL at time of sacrifice (4 months post-XRT) were determined by liquid chromatogra *Phys *tandem mass spectrometry (LC/MS/MS) as described earlier [21,22] using commercially available standards in 95% purity (Chromadex, Inc., Santa Ana, CA). Plasma flaxseed lignan levels were evaluated in 3 randomly selected mice per group (irradiated mice; diet initiated at -3, 0, +2, +4, +6 weeks of radiation exposure).

#### Bronchoalveolar lavage fluid analysis

Mice were sacrificed using an overdose of ketamine (100 mg/ml) and xylazine (20 mg/ml) at 4 months post-irradiation. Bronchoalveolar lavage (BAL) was then performed through a 20-gauge angiocatheter (BD Pharmingen, San Diego, CA), with the intra-tracheal instillation of 1 ml phosphate-buffered saline (PBS) containing an anti-protease cocktail (Sigma) and 5 mM EDTA given in 0.5 ml increments. An aliquot was immediately separated to measure total leukocyte cell counts (cells/ml BAL fluid) using a Coulter Cell and Particle Counter (Beckman Coulter, Miami, FL). The remaining lavage fluid was centrifuged at 1,200 rpm for 10 min and the cell-free supernatant was frozen at -80°C for cytokine and protein analysis.

The amount of total protein in the BAL fluid was assayed using the BCA Protein Assay Kit (Pierce, Rockford, IL) as per manufacturer's instructions. Absorbance was read at 560 nm (MRX Microplate Reader, Dynatech Laboratories, Chantilly, VA) and protein levels in mg/ml of BAL fluid were calculated.

### Multiplexed cytokine analysis

The cytokine concentrations in the BAL fluid were assayed using Invitrogen's Mouse Cytokine 20-Plex Panel (LMC0006). This panel permits simultaneous quantification of multiple cytokines in solution by capturing them onto antibody coated spectrally distinct fluorescent microspheres and measuring fluorescence intensity using the BioPlex 200 (Bio-Rad Laboratories, Hercules, CA) system. The kit quantified 20 mouse cytokines and chemokines: FGF-basic, Granulocyte Macrophage Colony-Stimulating Factor (GM-CSF), interferon-γ (IFN-γ), Interleukin (IL)-1α, IL-1β, IL-2, IL-4, IL-5, IL-6, IL-10, IL-12 (p40/p70), IL-13, IL-17, IP-10, keratinocyte-derived chemokine (KC), Monocyte Chemoattractant Protein-1 (MCP-1), Macrophage Inflammatory Protein-1α (MIP-1α), MIG, Tumor Necrosis Factor-α (TNF-α) and vascular endothelial growth factor (VEGF). The assay was performed according to the manufacturer's protocol. All the samples were run in duplicate. The detection limit of this kit is in pg/ml for all the included cytokines.

### Tissue harvesting and evaluation of oxidative lung injury

Radiation experiments were terminated after 16 weeks, corresponding to a time point when radiation-induced fibrosis is readily detectable in our model [10,21,22] using both biochemical assays and histopathological evaluation. For histological studies, the lungs were instilled prior to removal from the animal with 0.75 ml of buffered formalin through a 20-gauge angiocatheter placed in the trachea, immersed in buffered formalin overnight and processed for conventional paraffin histology. Sections were stained with hematoxylin and eosin and examined by light microscopy. Malondialdehyde (MDA), an indicator of oxidative stress [23] was measured in homogenized lung tissues using a commercially available kit (OXIS International, Portland, OR) according to manufacturer's protocol. The results were expressed as μmol MDA/g of lung protein.

### Oxygen saturation measurements

We used a mouse-adapted Pulse-oximeter (Starr Life Sciences, Oakmont, PA) as a non-invasive clinical readout of lung function post XRT in mice [24]. Mice were shaved around the neck area to remove black hair that interfered with detection and a pulse-oximeter clip was placed on the neck, over the carotid arteries. Mice were allowed to walk freely in a small chamber covered by a light blocking fabric supplied by the manufacturer. In some instances, mice were pre-adapted to the feeling of the neck sensor collar by adding a mock collar on them for a day prior to evaluation. Three minute readings were taken from each mouse, measurements containing error codes were removed and the average of the remaining readings was calculated (usually those ranged from 1000-3000). On a limited number of mice, arterial blood gas analysis of carotid blood was performed using an i-STAT blood gas analyzer (Abbott Laboratories, East Windsor, NJ) using G3+ cartridges and results compared to those from pulse oximetry.

### Quantitative and semi-quantitative assessment of fibrosis

Collagen content of mouse lung was evaluated quantitatively by determining hydroxy-proline (OH-Proline) content using acid hydrolysis [10] according to Woessner *et al*. [25]. The data is expressed as μg hydroxyproline/whole lung. Semi-quantitative evaluation of fibrosis was done histologically by determining a radiation Fibrotic Index (FI) as described previously [22].

### Statistical analysis

Results are expressed as mean ± SEM of two independent experiments. Statistical differences among groups were determined using one-way analysis of variance (ANOVA). When statistically significant differences were found (p < 0.05) individual comparisons were made using the Bonferoni/Dunn test (Statview 4.0). We used the Cox proportional-hazard model with a time-varying predictor to evaluate the effect of FS administration on animal survival (Proc Phreg; SAS Version 9.2; SAS Institute, Cary, NC).

## Results

### Detection of lignan content in murine plasma

Analytical methods (LC/MS/MS) were used to detect flaxseed metabolites in the circulation to confirm that mice fed on the flaxseed-supplemented diet were reaching *Phys*siologically relevant levels, comparable to those in all our previous studies [11,20,22]. Mice were maintained on control (0%FS) or treatment (10%FS) diets given *ad libitum *for either three weeks prior to XRT or started on same day of XRT or 2,4,6 weeks post XRT and continued for 4 months (see scheme on Figure 1). Plasma evaluation for lignan level detection was done at 16 weeks post XRT.

**Figure 2:**
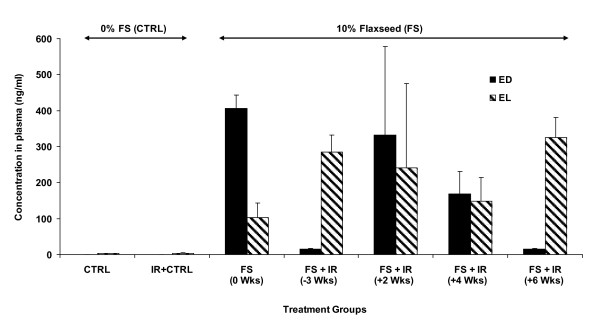
**Detection of flaxseed lignan metabolites in blood**. Circulating lignan (ED and EL) levels in plasma of mice, 4 months post-XRT, were determined using GC/MS/MS. Mice were fed with 0% or 10%FS diet initiated prior (-3 weeks) or post (+2, +4, +6 weeks) X-ray radiation therapy (XRT). Data is represented mean ± SEM (n = 3 mice per group).

Indeed (Figure 2), although variable, both ED and EL were detectable in all FS-fed mouse groups 4 months post-irradiation while no ED or EL was detectable in the control-diet fed mice. Levels of ED and EL ranged from 14-405 ng/ml and 103-325 ng/ml of plasma, respectively. These values fall within the reported range for our studies in other models of acute and chronic lung injury [11,20,22].

### Dietary flaxseed given post-XRT ameliorates radiation-induced cachexia and boosts survival

**Figure 3:**
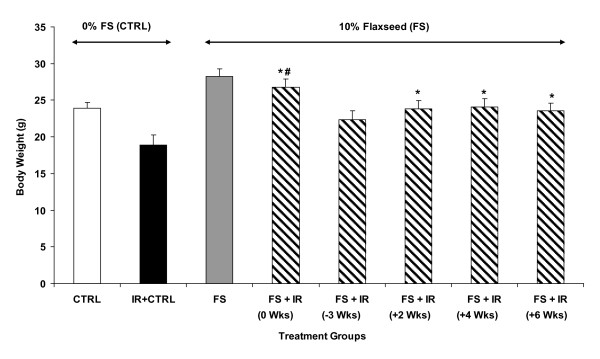
**Effect of Flaxseed (FS) diet on body weight of mice 4 months post-XRT**. Mice were fed with 0% or 10%FS diet prior (-3 weeks) or post (+2, +4, +6 weeks) X-ray radiation therapy (XRT). Body weight was recorded at 4 months post-irradiation. Data is represented mean ± SEM of two independent experiments (n = 15-25 mice per group). The white, black, gray and hatched bars represent untreated control, 0%FS + XRT, 10%FS and 10%FS + XRT groups, respectively. The dotted line represents weight average in non-irradiated control animals. *p ≤ 0.01 for irradiated 0%FS vs. irradiated 10%FS, ^$^p ≤0.01 for irradiated vs. non-irradiated 0% and #p ≤ 0.05 irradiated 10%FS (initiated 3 weeks prior to XRT) vs. all irradiated 10%FS (diet initiated on, or post-XRT).

**Figure 4:**
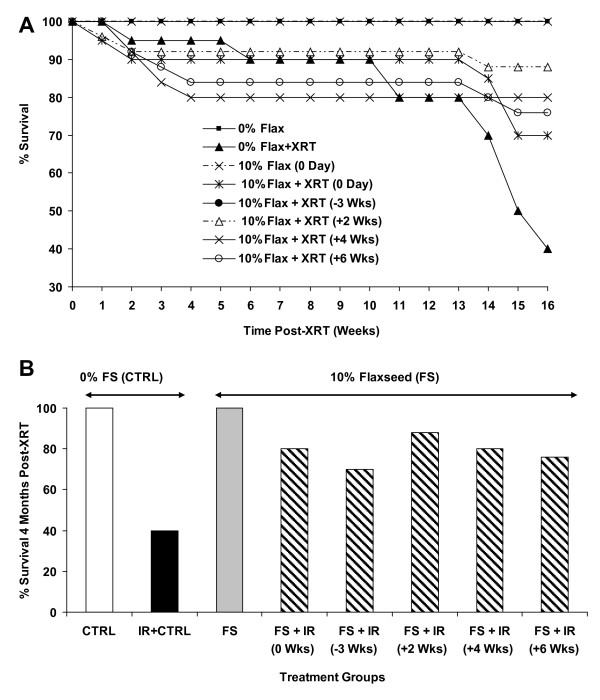
**Effect of Flaxseed (FS) diet on the survival of mice 4 months post-XRT**. Mice were fed with 0% or 10%FS diet prior (-3 weeks) or post (+2, +4, +6 weeks) X-ray radiation therapy (XRT) and observed for survival up to 16 weeks post-irradiation. Data is represented mean ± SEM of two independent experiments (n = 15 and 25 mice in non-irradiated and irradiated groups, respectively). **Panel A**: Kinetics of mouse survival. **Panel B: **Mouse survival 4 months post-XRT. The white, black, gray and hatched bars represent untreated control, 0%FS + XRT, 10%FS and 10%FS + XRT groups, respectively.

Radiation-induced pneumonopathy is associated with cachexia measured by loss in body weight. We therefore evaluated the effect of post-irradiation administration of 10%FS diet on XRT-induced loss in body weight (Figure 3) and survival (Figure 4) of mice. Although designed to be isocaloric, the 10%FS diet given to non-irradiated mice led to a significant (p < 0.007) difference in body weight (28.2 ± 1.07 g) as compared to non-irradiated mice on the 0%FS, control diet (23.8 ± 0.83 g). A fair comparison, therefore, of dietary treatments (0% vs. 10%) would be to compare each diet regimen with the non-irradiated counterparts. As anticipated, XRT decreased the body weight (18.9 ± 1.37 g) of animals on the 0% control diet significantly (p ≤ 0.01) as compared with their non-irradiated counterparts. Remarkably, the 10%FS supplementation, whether given 3 weeks prior to irradiation or several weeks thereafter, significantly prevented XRT-induced weight loss. The only exception was the condition where diet was initiated on the day of irradiation. In this group, the body weight was not significantly different than the 0%FS fed irradiated group.

XRT (13.5 Gy, 0%FS)-induced radiation sickness and early mortality was observed in all groups, albeit to variable degree, within the first 2-3 weeks of exposure (Figure 4A). We have observed this effect previously, and attributed this early death to poor oral intake possibly from esophagitis. Later deaths, occurring in 8-16 weeks are attributed to radiation pneumonopathy. However, by 4 months post XRT, only 40% of animals survived in the irradiated group (0%FS), as compared to the non-irradiated counterparts (Figure 4B). These values fall within the previously observed survival rate [22], given that the selected dose of 13.5 Gy reflects the LD50 as shown in our previous studies. Interestingly, 10%FS diet led to a significant (p < 0.05) increase in the survival of animals in all groups when compared with irradiated group (XRT, 0%FS). When 10%FS diet was started preventively, i.e., 3 weeks prior to XRT, survival was enhanced significantly (80%; p < 0.05) in comparison to irradiated group, a finding in agreement with previous observations. Remarkably, flaxseed diet, when given therapeutically, i.e., initiated post-XRT, enhanced the survival of all irradiated animal groups. When 10%FS diet was given to animals up to 6 weeks post-irradiation, animal survival increased significantly (p < 0.05).

### Dietary FS given post-XRT improves pulmonary hemodynamics and mitigates pulmonary inflammation and oxidative lung injury

Thoracic radiation is associated with pneumonopathy characterized by inflammatory cell influx and pulmonary edema. We have previously shown that preventive use of dietary FS decreased radiation-induced lung damage and inflammation [22]. To test whether FS also mitigates these effects when administered at variable times post radiation challenge, we delayed the dietary administration (0, 2, 4, 6 weeks) and evaluated BAL at 4 months post-XRT. We confirmed BAL findings with a histopathological evaluation of lungs in mice belonging in the same groups of mice, which, however, were not lavaged, so that histological evaluation would not be compromised.

**Figure 5:**
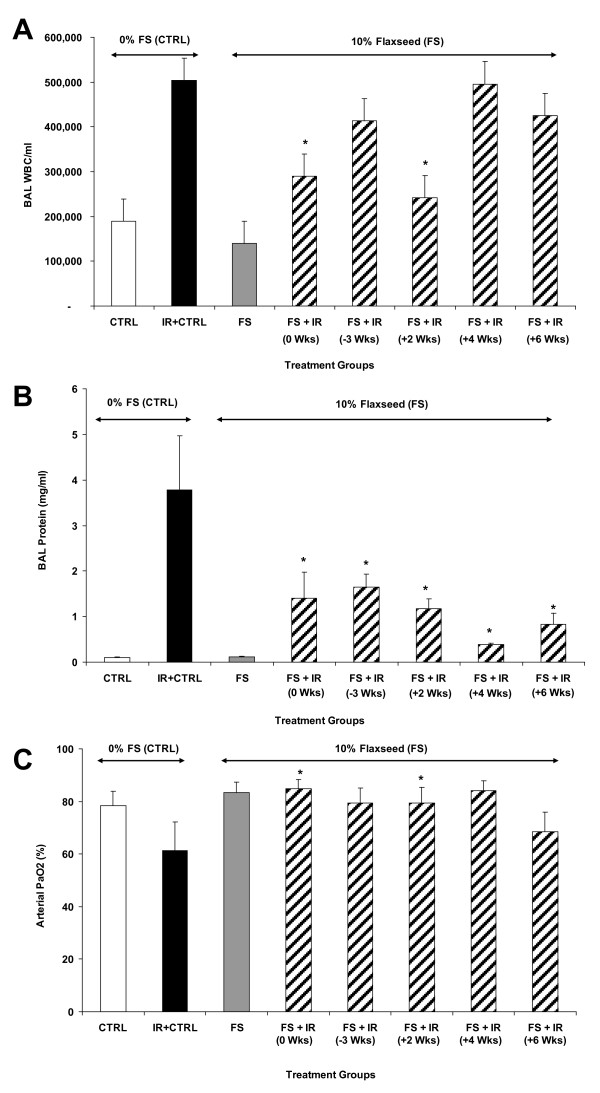
**Evaluation of Lung Injury, Inflammation and Blood Oxygenation Levels in mice 4 months post-XRT**. Mice were fed with 0% or 10%FS diet at designated times (-3, 0, +2, +4, +6 weeks) of X-ray radiation therapy (XRT). Data is represented mean ± SEM of two independent experiments (n = 15-25 mice per group). **Panel A: **Total WBC counts in bronchoalveolar lavage (BAL) of irradiated (13.5 Gy, XRT) mice after 4 months. *p ≤ 0.05 for irradiated 0%FS vs. irradiated 10%FS **Panel B: **Total proteins in BAL of mice after 4 months. *p ≤ 0.01 for irradiated 0%FS vs. irradiated 10%FS **Panel C: **Arterial O_2 _levels were measured using pulse oximetry in irradiated (13.5 Gy, XRT) mice after 4 months. The white, black, gray and hatched bars represent untreated control, 0%FS + XRT, 10%FS and 10%FS + XRT groups, respectively. *p ≤ 0.05 for irradiated 0%FS vs. irradiated 10%FS.

**Figure 6:**
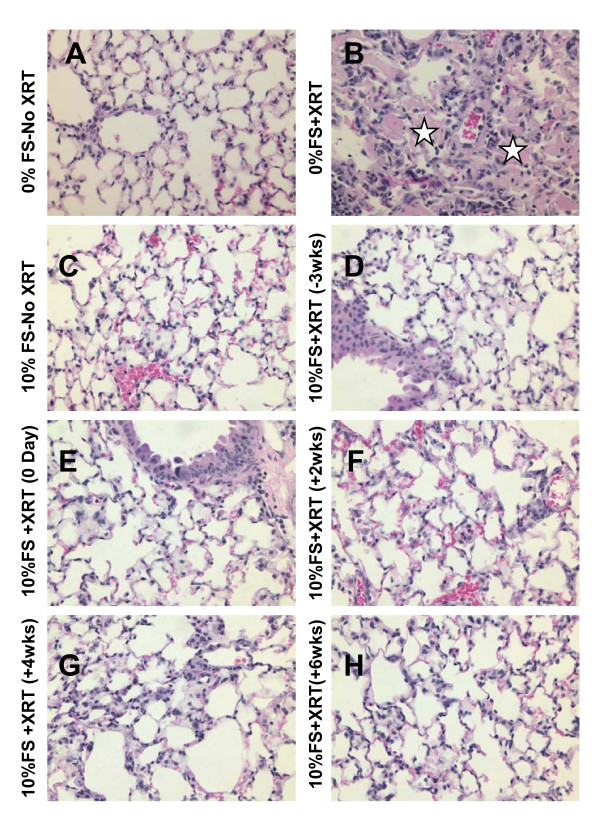
**Histological evaluation of lung Hematoxylin and Eosin (H&E)-stained sections post-XRT (4 months)**. Mice were fed with 0% or 10%FS diet prior (-3 weeks) or post (+2, +4, +6 weeks) X-ray radiation treatment (XRT). Lungs were harvested 4 months post single fraction XRT and processed for histology. Asterisks designate proteinaceous exudate in alveolar spaces.

We observed a significantly reduced inflammatory cell influx (Figure 5A) in the BAL fluid from lungs in all irradiated mouse groups fed with 10%FS diet as compared with those on control diet, regardless of the time the diet was initiated (pre- or post-XRT). Alveolar protein level is a marker for increased lung permeability and lung injury. Alveolar protein exudate was increased 37-fold (3.78 ± 1.19 mg/ml) in mice 4 months post-XRT (Figure 5B) in comparison to untreated controls (0.1 ± 0.013 mg/ml). These findings correlated with the histopathological evaluation (Figure 6). In summary, FS diet significantly (p < 0.05) decreased lung injury and edema by decreasing the XRT-induced alveolar protein levels irrespective of therapeutic or preventive administration.

Radiation pneumonopathy is also associated with compromised pulmonary function resulting in poor oxygenation levels as modeled in rodent models [26]. Pulse oximetry is a non-invasive way of determining arterial blood oxygenation levels (Figure 5C). XRT notably decreased the percentage of arterial O_2 _levels (61.4% ± 10.77%) in mice fed with 0% control diet in comparison to untreated control animals (78.5% ± 5.33%). Mice Fed with 10%FS diet had an increase percentage of arterial O_2 _levels compared to mice fed 0% diet following XRT. FS diet enhanced blood oxygenation, thus improving pulmonary hemodynamics in irradiated mice irrespective of whether it was given therapeutically or preventively.

### Dietary FS mitigates oxidative lung changes when given post-thoracic XRT

**Figure 7:**
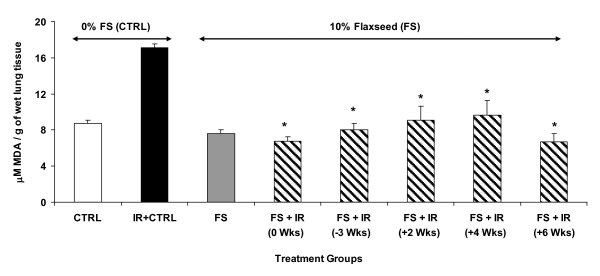
**Effect of 10%FS diet on lung lipid peroxidation levels 4 months post-XRT**. Mice were fed with 0% or 10%FS diet prior (-3 weeks) or post (+2, +4, +6 weeks) X-ray radiation therapy (XRT). Lungs were harvested after 4 months, homogenized and assayed for lipid peroxidation by measuring Malondialdehyde (MDA) levels. MDA is calculated per g of wet lung tissue. Data is represented mean ± SEM of two independent experiments (n = 15-25 mice per group). The white, black, gray and hatched bars represent untreated control, 0%FS + XRT, 10%FS and 10%FS + XRT groups, respectively. *p ≤ 0.0002 for irradiated 0%FS vs. irradiated 10%FS.

Lipid peroxidation plays a major role in mediating oxidative-damage in tissues. Thoracic radiation-induced oxidative degradation of unsaturated fatty acids can be followed by determining the amount of a product, malondialdehyde (MDA), of lipid peroxidation in lung tissues [21,22]. A significant two-fold increase in MDA levels was recorded in irradiated animals fed with control diet as compared to unchallenged controls (Figure 7). In contrast, mice fed with a 10%FS diet (whether given preventively or therapeutically) maintained a significantly (p ≤ 0.005) lower MDA level at 4 months post-XRT in all the treatment groups (diet initiated -3 or 0,2,4,6 weeks post XRT) as compared to irradiated mice fed with 0%FS diet. Importantly, no statistical difference was found among any of the irradiated FS-fed groups (Figure 7, hatched bars) and the non-irradiated FS group (Figure 7, grey bar).

### Dietary FS mitigates fibrotic changes in lung tissue when given post-thoracic XRT

**Figure 8:**
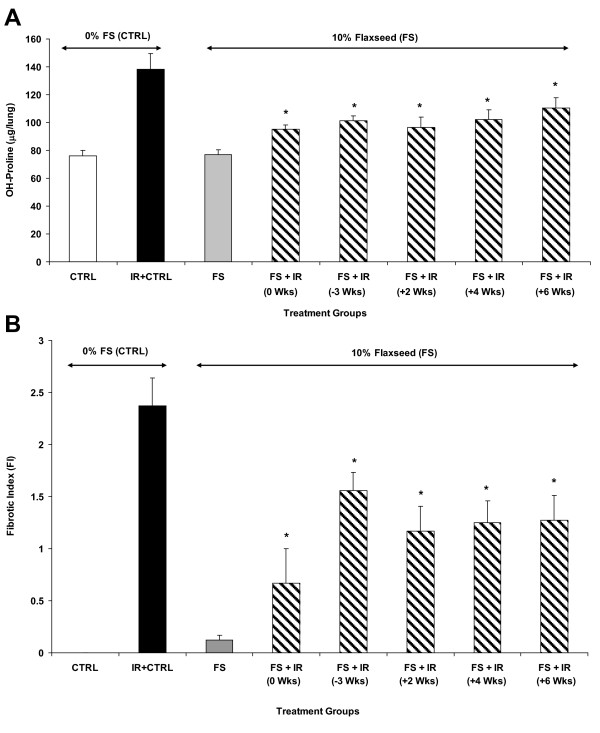
**Determination of fibrotic changes in mouse lungs 4 months post-XRT**. **Panel A: **Evaluation of OH-Proline content in lungs of irradiated (13.5 Gy, XRT) mice. Mice were fed with 0% or 10%FS diet prior (-3 weeks) or post (+2, +4, +6 weeks) XRT. Lungs were harvested after 4 months and OH-Proline assay was performed. *p ≤ 0.005 for irradiated 0%FS vs. irradiated 10%FS. **Panel B: **Fibrotic Index range = 0 - 4). Data is represented mean ± SEM of two independent experiments (n = 15-25 mice per group). The white, black, gray and hatched bars represent untreated control, 0%FS + XRT, 10%FS and 10%FS + XRT groups respectively. *p ≤ 0.005 for irradiated 0%FS vs. irradiated 10%FS. *p ≤ 0.005 for irradiated 0%FS vs. irradiated 10%FS.

Dietary flaxseed prevents radiation-induced pulmonary fibrosis in mice when given preventively [22]. However, its effects when given therapeutically, i.e., post the radiation challenge, are unknown. To test this, we evaluated the total OH-Proline content of murine lungs 4 months post-XRT (Figure 8A). This is a quantitative measure of collagen deposition and fibrosis in lungs, which is expressed as μg OH-proline/lung. XRT led to a significant (p ≤ 0.0001), near 2-fold increase of OH-Proline in mice (0%FS) in comparison to unchallenged controls. FS diet rendered a noteworthy decline in OH-Proline levels in all radiation-challenged mice. Mice fed with 10%FS diet prior or post-XRT had significantly (p ≤ 0.005) deceased levels of OH-Proline content in comparison to those fed with 0%FS following XRT (Figure 8A).

**Figure 9:**
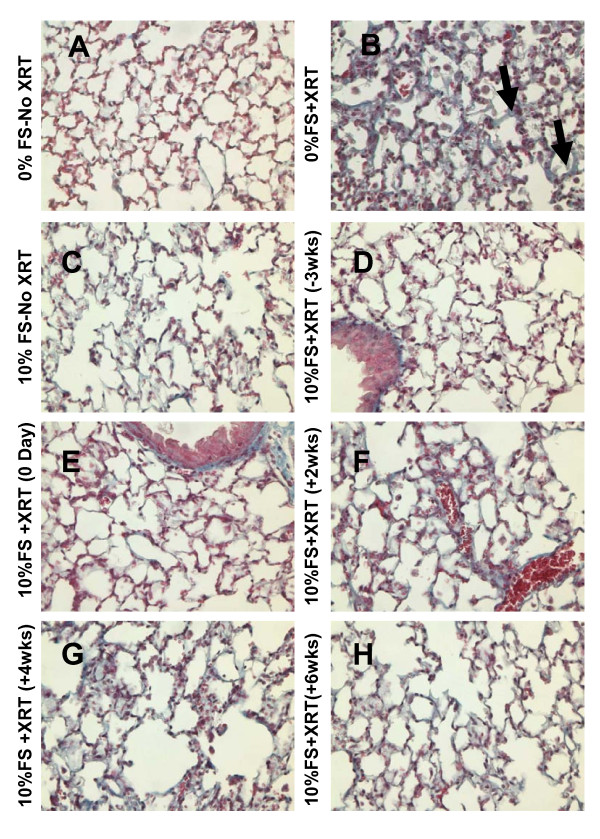
**Histological evaluation of lung Trichrome-stained sections post-XRT (4 months)**. Mice were fed with 0% or 10%FS diet prior (-3 weeks) or post (+2, +4, +6 weeks) XRT. Lungs were harvested 4 months post single fraction XRT and processed for histology. Arrows designate collagen deposition in lung (blue color).

To further evaluate the degree of XRT-induced fibrosis and correlate biochemical findings with tissue histology, lungs were also examined histolopathologically using Trichrome staining (Figure 9) and Fibrotic Index (FI) score, a measure of the extent of lung fibrosis and inflammation, was determined for each lung (Figure 8B). Lungs of irradiated mice fed with 0%FS diet showed severe fibrosis (Figure 9B, blue color) as compared to non-irradiated mice (0%FS). The extent of fibrosis was notably reduced in all animal groups fed with 10%FS diet prior or post-XRT when compared to irradiated 0%FS fed mice.

### Alterations of pro-inflammatory and pro-fibrotic cytokine profile in the BAL fluid of FS-fed mice 4 months post-XRT

Thoracic radiation-induced cytokines, chemokines and cell adhesion molecules are implicated in the pathogenesis of pulmonary inflammation, both subacute and chronic, as well as in the development of lung fibrosis [27]. To determine the beneficial effects of FS given several weeks post radiation challenge, we evaluated inflammatory cytokine levels in BAL fluid in mice sacrificed 4 months post XRT (Table 1). This time coincides with radiation pneumonitis and the onset of detectable lung fibrosis. Using multiplex cytokine analysis, we analyzed the effect of FS on a panel of 20 cytokines. While half the cytokines (basic FGF, GM-CSF, IFN-γ, IL-1α, IL-5, MCP-1, TNF-α) were at levels below the detection limit of the assay, 10 cytokines (IL1β, IL-2, IL-4, IL-6, IL-12, IL-17, IP-10, KC, MIG, MIP-α and VEGF) were measurable in the BAL fluid of all mouse groups.

**Table 1:** Evaluation of cytokine levels in BAL of mice 4 months post-XRT.

Treatment Groups	IL-1β	IL-4	IL-6	IL-12 (p40/p70)	IL-17	KC	MIG	MIP-1α	VEGF
**0%FS**	26.7 ± 8.3	28.6 ± 6.3	21.0 ± 5.9	19.2 ± 3.1	0.6 ± 0.2	658.6 ± 39.3	15.3 ± 2.5	25.4 ± 7.8	26.6 ± 4.6
**0%FS + XRT**	12.1 ± 2.7	17.4 ± 1.0	66.0 ± 42.5	88.1 ± 18.2	0.4 ± 0.1	173.0 ± 47.0	11.4 ± 4.2	18.5 ± 3.7	43.9 ± 4.5
**10%FS**	14.4 ± 5.1	20.4 ± 2.3	16.6 ± 2.0	12.9 ± 1.3	0.4 ± 0.1	730.7 ± 58.4	14.6 ± 4.4	16.5 ± 3.0	21.1 ± 2.9**^#^**
**10%FS + XRT (0 Day)**	12.4 ± 0.8	18.2 ± 0.3	20.4 ± 1.1**^$^**	51.1 ± 13.3**^$^**	0.6 ± 0.1	521.0 ± 165.0*	20.9 ± 4.9	13.3 ± 1.5	21.4 ± 3.8**^#^**
**10%FS + XRT (-3 wks)**	14.7 ± 1.5	17.9 ± 0.6	28.0 ± 6.4	54.2 ± 13.6*	0.9 ± 0.3*	396.0 ± 49.3	14.8 ± 2.8	15.7 ± 1.6	16.9 ± 3.4**^#^**
**10%FS + XRT (+2 wks)**	13.1 ± 2.9	15.9 ± 1.0	16.6 ± 1.7*	43.2 ± 7.9*	0.3 ± 0.1**^§§^**	380.5 ± 162.7	7.2 ± 2.0	14.5 ± 1.7	13.4 ± 1.3**^#^**
**10%FS + XRT (+4 wks)**	7.4 ± 1.0	16.6 ± 0.6	13.0 ± 1.8*	16.2 ± 1.2**^# §^**	0.1 ± 0.0**^§§^**	444.0 ± 66.8	8.8 ± 1.6	10.0 ± 1.2	6.8 ± 2.2**^#^**
**10%FS + XRT (+6 wks)**	7.7 ± 1.2	16.5 ± 0.9	15.3 ± 1.7**^$^**	26.8 ± 7.1**^#^**	0.2 ± 0.0**^§§^**	264.7 ± 150.7	9.0 ± 4.8	10.0 ± 2.1	11.7 ± 1.8**^#^**

Mice were fed with 0% or 10%FS diet prior (-3 weeks) or post (+2, +4, +6 weeks) X-ray radiation therapy (XRT) and sacrificed after 4 months. Lungs were lavaged and cytokine levels in BAL were measured. Data is represented mean ± SEM of two independent experiments (n = 15 and 25 mice in non-irradiated and irradiated groups, respectively). Values are mean ± SEM of two independent experiments. n = 15 and 25 in non irradiated and irradiated groups respectively.

**^$^**p ≤ 0.05, *p ≤ 0.01, **^#^**p ≤ 0.0005, for 0%FS irradiated vs. 10%FS irradiated groups.

**^§^**p ≤ 0.05, **^§§ ^**p ≤ 0.01 for 10%FS given preventively to XRT Vs 10%FS started therapeutically post-XRT.

A significant decrease in the levels of IL-1β, IL-2, IL-4, MIG, or MIP-1α was detected in all of the irradiated FS diet fed groups as compared to irradiated mice on control diet. However, levels of IL-6, IL-12, IL-17 and VEGF were significantly lower in irradiated FS-fed mice as compared to irradiated mice on control diet. Notably, even delaying FS diet by as long as 6 weeks post-radiation challenge resulted in a several-fold decrease of the above-mentioned cytokines.

## Discussion

We demonstrate here for the first time the role of FS in boosting survival and mitigating the acute and chronic damage induced by X-ray radiation exposure of lung tissues when administered days and even weeks after radiation exposure. Results from our study show that FS significantly ameliorates the XRT-induced damage by improving survival and body weight of mice fed with FS not only when diet was given prior to XRT but also when diet was started 2, 4 and 6 weeks after XRT. We also found that FS diet mitigated the deleterious effects of XRT by: a) improving pulmonary hemodynamics and blood oxygenation levels, b) decreasing lung injury by lowering BAL protein levels, c) reducing pulmonary fibrosis by decreasing collagen content of lung tissues, d) reducing lung inflammation by decreasing WBC influx into the airways and by e) oxidative modification of mouse lungs as evidenced by levels of lipid peroxidation. BAL cytokine analysis, moreover, pointed to an alteration of the chronic inflammatory profile of irradiated lungs favoring a mitigated radiation effect as a result of the FS diet.

Some reports suggest using lung lavage to remove radionuclides inhaled after a dirty bomb detonation as a possible countermeasure [28,29]. However, this remains an impractical countermeasure since multiple lavages may be required for efficient removal of radionuclide burden while the procedure itself is associated with known risks of an invasive procedure that requires anesthesia. Alternatively, a plethora of compounds both chemical/natural are being evaluated with the intent of mitigating radiation damage [17]. To date Amifostine is the only FDA approved cytoprotective radiation mitigator. However, the use of Amifostine has been limited by its significant systemic toxicity [17]. Further, most of the compounds that offered a positive radioprotection on cells have not shown efficacy in pre-clinical animal studies.

An ideal radiation mitigator should be safe, effective, have a long shelf life and an easy route of administration. Flaxseed, due to its high content of lignans and omega-3 fatty acids, is a dietary supplement that has numerous medicinal, anti-inflammatory and antioxidant properties. FS and its bioactive components have been extensively studied for their anti-inflammatory [11,20], anticarcinogenic [30,31] and anti-atherogenic effects [32] in several organ systems. Importantly, prolonged FS administration has not been associated with any significant toxicity [33]. Therefore, we hypothesized that flaxseed may be an effective, safe and cost-effective mitigator of the radiation damage.

Our data showed that 10%FS diet supplementation significantly increased the survival in mice in all the irradiated groups (Figure 4) irrespective of the time of initiation of the FS diet (70-88% survival) as compared to the survival of mice fed with 0%FS diet (40% survival). It is evident from our results that FS diet protects mice from XRT-induced mortality whether given therapeutically or preventively. Improvement of survival using antioxidants such as N-acetyl-Cysteine (NAC) or mitochondrion-targeted small molecule radiation damage mitigators has been shown in mouse models of abdominal irradiation [34] or total body irradiation [35] respectively. To our knowledge our study is the first to report that a mitigator of radiation damage improves survival of animals in an experimental model of thoracic radiation damage. Results revealed radiation-induced increment in lipid peroxidation in lungs (Figure 7). Lipid peroxidation (LP) results from a cascade of events induced by radiation in biological membranes. FS diet led to a significant drop off in the LP levels in all the FS-fed experimental mouse groups. Some reports show that other plant extracts also decrease radiation-induced LP [36]. Recent work by Gauter-Fleckenstein *et al*. [37] showed mitigation of radiation-induced oxidative lung tissue changes by an superoxide dismutase (SOD) mimetic, although beneficial effects of the mitigator were limited. Oxidative stress in lungs was only then mitigated when the SOD mimetic was given up to 24 hours post radiation and not when given days (3 days) or weeks (8 weeks) post irradiation. In our work the drop in lipid peroxidation of lung tissues was significant even when the FS diet was initiated 4 and 6 weeks post radiation challenge. Further, our lab is currently exploring dietary formulations of flaxseed components, to identify the chief bioactive ingredient(s) that mitigate radiation effects.

Radiation-induced inflammation is an important side effect that contributes to normal tissue injury [7]. Our results indicated that XRT-induced lung inflammation and impaired blood oxygenation (decreased P_a_O2) were improved with FS diet when initiated prior to or, importantly, days and weeks after XRT. Vujaskovic and coworkers [38] have shown that severe hypoxia develops months post an initial radiation exposure of lung tissues. Such hypoxia contributes significantly to the development of a cascade of events leading to lung injury. Improved blood oxygenation of all FS-fed mouse groups (Figure 5C) may lead to decreased levels of tissue hypoxia and may thus, explain mitigation of adverse radiation effects even when diets are initiated post challenge. In fact, recent work by the same group [37] using an SOD mimetic to mitigate tissue hypoxia, showed that this would be possible even when given weeks post-irradiation, something which further corroborates with our findings with the FS diet. Decrease of radiation-induced lung inflammation by a mitigator has never been shown. This is the first report that antioxidant agents, such as the FS diets, mitigate pulmonary inflammation when given weeks post initial challenge.

A major feature of radiation pneumonitis is a considerable increase in the alveolar protein exudates, an indicator of increased vascular permeability and direct lung injury [38]. Radiation causes damage to resident lung cells which in turn release inflammatory mediators (cytokines) and recruit inflammatory cells to the site [39]. BAL protein level is the most direct and reliable measure of lung injury, translating in actual tissue damage while activation markers of lung tissue inflammatory cell content such as the macrophage ED-1 marker, is a measure rather of inflammation and not injury [37]. Our results show a significant mitigation of lung injury in all the experimental FS diet fed groups, regardless of the time of FS diet initiation. This may be attributed to decreased inflammatory cell influx and membrane oxidation in FS-supplemented mice. This is the first report that an agent is reported to mitigate *Phys*siological lung injury from radiation *in vivo*.

Radiation pneumonitis also involves irreversible fibrotic changes in lung tissues occurring in the late phase of the radiation response [40]. We have shown that wholegrain FS was protective against experimental radiation fibrosis [22]. Our current study showed for the first time that fibrotic processes can be blunted in pulmonary tissues even when the protective agent is given post-radiation damage, i.e., as a radiation mitigator. Despite notable benefits of a therapeutic usefulness of FS diet (i.e., when initiated at 0, +2, +4 and +6 weeks post XRT), however, the fact remained that FS-mediated decline in both lung OH-Proline levels and FI was more prominent when diet was started preventively, i.e., 3 weeks prior to XRT. This is the first time that any botanical or chemical agent is indicating mitigation of fibrotic changes effect in lungs.

Radiation exposure leads to histologically recognizable chronic injury initiated by a series of molecular responses involving a number of inflammatory cytokines, pro-fibrotic cytokines and chemokines produced by a variety of cell types, including macrophages, epithelial cells and fibroblasts [27,39,41]. While many studies in rodent thoracic irradiation models focus on cytokine release during the initial post-irradiation phase [41], studies such as those of Rubin *et al*. [39] analyzed the cytokine profile in lungs during the late, chronic phase. In the present study, we evaluated the effect of FS diet on inflammatory cytokines in the late phase of thoracic radiation-exposed mice. As expected, the cytokine levels of the lung tissues and fluids measured in our model reflect levels comparable to those in chronic inflammation and not in acute responses. Several cytokines associated with inflammation were significantly lower in irradiated FS-fed mice as compared to irradiated mice on control diet, while no other cytokines were significantly aggravated by the FS diet. Our studies show, for example, a sustained 3-fold increased BAL level of IL-6 in radiation-exposed mice if fed a control diet while dietary supplementation of flaxseed, given even weeks post initial insult, decreased IL-6 levels, reflecting a low inflammatory state of lung tissues.

Radiation-induced pulmonary injury consists of an early latent period followed by chronic inflammation that leads to collagen deposition and ultimately to fibrosis. Studies suggest that chronic inflammation occurs due to the release of cytokines, chemokines, growth factors and it induces the development of fibrosis. Rubin *et al*. [39] reported that a perpetual cascade of cytokines activated soon after radiation exposure that leads to late pulmonary fibrosis.

In the present study we also evaluated the effect of FS diet on inflammatory cytokines in radiation-exposed murine models. Macrophage inflammatory protein 1-α (MIP-1α) appears to be an important cytokine mediator of pulmonary inflammation and injury. Growing pre-clinical and clinical data suggest a potential relationship between serum MIP-1α levels and the risk of lung injury following thoracic radiation.

Increased production of IL-6 is known to occur in the chronic inflammation scenario. It stops production of TNF-α, an acute phase inflammatory cytokine, and is found in chronic disease conditions like thyroditis and type I diabetes. Our studies also show a high level of IL-6 in radiation-exposed mice. Dietary supplementation of flaxseed decreased IL-6 levels, indicating a low inflammatory state. Both IL-4 and IL-17 are known to induce IL-6 production. However in our study, the increase in IL-6 in radiation exposed mice cannot be attributed to IL-17 and IL-4 as both these cytokines showed lower expression as compared to untreated controls.

IL-12 is an inducer of cell mediated inflammation. In our studies, decreased IL-4 expression in radiation exposed mice was concomitant with increase in IL-12 expression. This antagonistic nature of IL-4 and IL-12 is well reported in literature. When flaxseed was given therapeutically or preventively to irradiated mice, it led to a decrease in IL-12 expression without changing IL-4 status. Flaxseed thus seems to decrease cell mediated inflammation by decreasing IL-12 levels although it does not have any effect on IL-4.

VEGF is identified as an endothelial cell specific growth factor that contributes to angiogenesis and vascular permeability. Radiation exposure to lungs induces hypoxia [42] and hypoxia itself induces ROS generation which in turn promotes inflammation and vascular damage, activates pro-fibrotic cytokines, and promotes collagen formation. Our results corroborate these reports, as we observed increased VEGF levels in the BAL fluid of radiation-exposed mice, while in FS-fed irradiated animals low VEGF levels were observed as compared to untreated controls.

## Conclusion

Because radiation damage is a multi-faceted phenomenon, any agent or compound that can modify or alter multiple aspects or mechanisms of radiation-induced inflammation and fibrosis while at the same time being both inexpensive and non-toxic is extremely exciting. It is evident from our findings that dietary FS is a potential agent in mitigating radiation damage and that the discovery of the mitigating properties of FS may prove a critical milestone in the development of non-toxic radiation mitigators.

## List of abbreviations

AU: Arbitrary units; BAL F: Bronchoalveolar lavage fluid; ED: Enterolactone; EL: Enterodiol; FI: Fibrotic Index; FS: Flaxseed; H&E: Hematoxylin and eosin; IR: Irradiation; OH-Proline: Hydroxy-proline; LP: Lipid Peroxidation; MDA: Malondialdehyde; PMN: Polymorphonuclear leukocyte; ROS: Reactive oxygen species; SDG: Secoisolariciresinol diglucoside; SEM: Standard error means; WBC: White blood cells; XRT: X-ray Treatment.

## Competing interests

The authors declare that they have no competing interests.

## Authors' contributions

MCS designed the study and individual experiments, analyzed data, wrote the manuscript and supervised lab personnel. ST assisted with animal experiments and manuscript preparation. KT performed statistical analysis of survival studies. SH performed all the irradiation procedures. RP performed animal experiments, biochemical assays and conducted data analysis. FD assisted with pulse oximetry. EA conducted animal experiments and tissue analyses. DFH performed statistical analysis of survival studies. CCS performed pathology assessment of histological specimens. KAC assisted with irradiation procedures and provided consultation on data analysis. All authors read and approved the final manuscript.

## Pre-publication history

The pre-publication history for this paper can be accessed here:

http://www.biomedcentral.com/1471-2407/11/269/prepub
